# Supplementary material for: Study on pharmacological properties and cell absorption metabolism of novel daidzein napsylates
Source: R Soc Open Sci. 2021 Jan 13;8(1):201475. doi: 10.1098/rsos.201475 (PMC7890489; doi:10.1098/rsos.201475)
Supplement: Tables about RSD & RE and Figures about MS [file rsos201475supp1.docx]

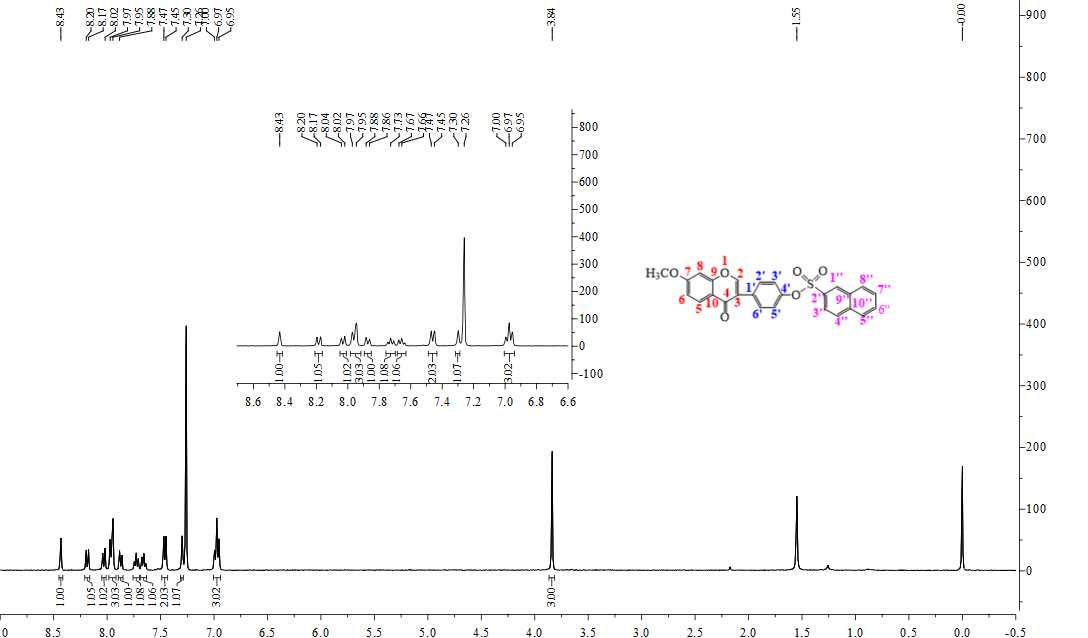


^1^H NMR (400 MHz, CDCl_3_) δ 8.43 (s, 1H, C1"-H), 8.18 (d, *J* = 8.8 Hz, 1H, C5-H), 8.03 (d, *J* = 8.8 Hz, 1H, C4"-H), 7.97 – 7.95 (m, 3H, C2-H, C2'-H, C6'-H), 7.87 (d, *J* = 8.7 Hz, 1H, C3"-H), 7.73 (t, *J* = 7.3 Hz, 1H, C7"-H), 7.66 (t, *J* = 7.3 Hz, 1H, C6"-H), 7.46 (d, *J* = 7.9 Hz, 2H, C5"-H, C8"-H), 7.30 (s, 1H, C8-H), 7.00 – 6.95 (m, 3H, C6-H, C3'-H, C5'-H), 3.84 (s, 3H, 7-OCH_3_).

1H-NMR spectrum of DD4


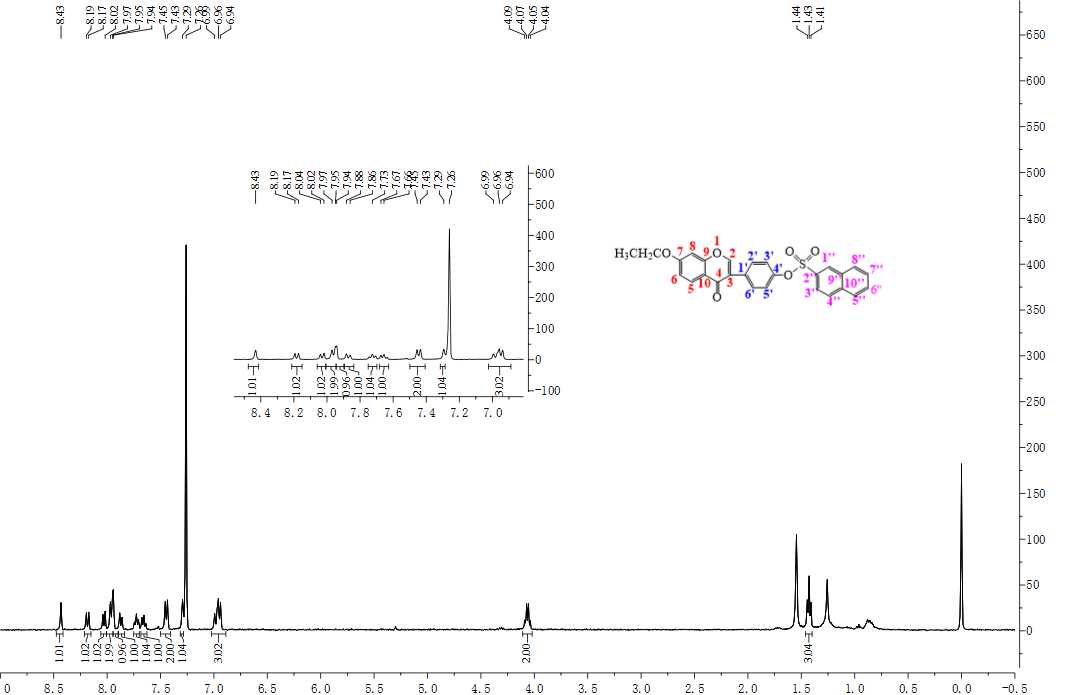


^1^H NMR (400 MHz, CDCl_3_) δ: 8.43 (s, 1H, C1"-H), 8.18 (d, *J* = 8.6 Hz, 1H, C5-H), 8.03 (d, *J* = 8.5 Hz, 1H, C4"-H), 7.96 (d, *J* = 8.5 Hz, 2H, C2'-H, C6'-H), 7.94 (s, 1H, C2-H), 7.87 (d, *J* = 8.7 Hz, 1H, C3"-H), 7.73 (t, *J* = 7.4 Hz, 1H, C7"-H), 7.66 (t, *J* = 7.6 Hz, 1H, C6"-H), 7.44 (d, *J* = 8.1 Hz, 2H, C5"-H, C8"-H), 7.29 (s, 1H, C8-H), 6.99 – 6.94 (m, 3H, C6-H, C3'-H, C5'-H), 4.06 (q, *J* = 7.0 Hz, 1H, 7-OCH_2_-), 1.43 (t, *J* = 6.9 Hz, 1H, -CH_3_).

1H-NMR spectrum of DD5
