# Supplementary material for: Study on pharmacological properties and cell absorption metabolism of novel daidzein napsylates
Source: R Soc Open Sci. 2021 Jan 13;8(1):201475. doi: 10.1098/rsos.201475 (PMC7890489; doi:10.1098/rsos.201475)
Supplement: Experimental data [file rsos201475supp2.pdf]

Certificate of English Language Editing

Manuscript Title:

Study on Pharmacological Properties and Cell Absorption Metabolism of Novel Daidzein Napsylates

Date of Revision:

October 21, 2020

Abstract:

Novel daidzein napsylates (DD4 and DD5) were synthesised by microwave irradiation, according to structural modification of daidzein (DAI) using the principle of pharmacokinetic transformation. The pharmacological properties of DD4 and DD5 were evaluated via HPLC and cDalculated based on the drug design software ChemAxon 16.1.18. The pharmaceutical properties and cell uptake changes of DD4 and DD5 were investigated to analyse the structure–property relationship. The metabolisms of DD4 and DD5 were analysed by HPLC-MS in human aortic vascular smooth muscle cells and their possible metabolic pathways were inferred in vitro. The results showed that the solubility of DD4 and DD5 was increased by 2.79×105 and 2.16×105 times compared to that of DAI, separately, in ethyl acetate. The maximum absorption rates of DD4 and DD5 were 87.97% and 90.34% in human aortic vascular smooth muscle cells (HAVSMCs) with the order of DD5≈DD4>DAI, which demonstrated that maximum cell absorption rates of DD4 and DD5 were enhanced by 4.3–4.5 times relative to DAI. Preliminary studies on metabolites of DD4 and DD5 in HAVSMCs showed that DD4 and DD5 were hydrolysed into DAI under the action of intracellular hydrolase in two ways, ester hydrolysis or ether hydrolysis. Then, DAI was combined with glucuronic acid to form daidzein monoglucuronate under the action of UDP-glucuronidase. Meanwhile, it was also found that metabolite M5 of DD5 could...

This document certifies that the manuscript listed above was copy edited for proper English language at LetPub. All of our language editors are native English speakers with long-term experience in editing scientific and technical manuscripts. We are committed to leveling the playing field for researchers whose native language is not English.

- Neither the research content nor the authors' intended meaning were altered in any way during the editing process.
- Documents receiving this certification should be considered ready for publication where language issues are concerned. *However, the authors may accept or reject LetPub's suggestions and changes at their own discretion.*
- If you have any questions or concerns about this edited document, please contact us at support@letpub.com

LetPub
Your Manuscript Editor

LetPub is an author service brand owned and operated by Accdon LLC. Headquartered in the Boston area, we are a full-spectrum author services company with a large team of US-based certified language and scientific editors, ISO 17001 accredited translators, and professional scientific illustrators and animators. We advocate ethical publication practices and are an official member of the Committee on Publication Ethics (COPE).

For more information about our company, services, and partnership programs, please visit www.letpub.com.

© 2020 Accdon, LLC. All Rights Reserved. Tel: 1-781-202-9968 Email: info@accdon.com Address: 400 Fifth Ave, Suite 530, Waltham, MA 02451, United States
